# Postgraduate students’ perceptions of artificial intelligence integration in research: A cross-sectional study

**DOI:** 10.1371/journal.pone.0345726

**Published:** 2026-03-24

**Authors:** Ibrahim Naif Alenezi, Fathia Ahmed Mersal, Amal Ahmed Elbilgahy

**Affiliations:** 1 Faculty of Nursing, Northern Border University, Public Health Nursing Department, Arar, Saudi Arabia; 2 Public Health Nursing Department, Faculty of Nursing Northern Border University, Arar, Saudi Arabia; 3 Maternal & Child Health Nursing Department, Faculty of Nursing, Northern Border University, Arar, Saudi Arabia; University of Basel Institute for Biomedical Ethics: Universitat Basel Institut fur Bio- und Medizinethik, SWITZERLAND

## Abstract

**Background:**

Generative artificial intelligence (AI) tools such as ChatGPT are increasingly used in academic research, yet evidence on postgraduate students’ perceptions remains limited in non-Western and health-professional contexts. Understanding how students perceive AI’s benefits, risks, and ethical implications is essential for informing institutional research policies.

**Methods:**

This cross-sectional case study surveyed 267 master’s students enrolled in nursing and health profession programs at Northern Border University in Arar, Saudi Arabia. Data were collected between October 1 and November 15, 2025, using a validated 54-item questionnaire that assessed perceived benefits, perceived risks, privacy concerns, mistrust in AI, performance anxiety, social bias, regulatory matters, liability issues, and intention to adopt AI tools. Multiple linear regression with heteroscedasticity-robust (HC3) standard errors was used to identify predictors of AI adoption intention.

**Results:**

Most participants (85.0%) reported prior use of AI tools, predominantly ChatGPT. Perceived benefits were the strongest predictor of intention to adopt AI for research purposes (β = 0.588, p < 0.001). Privacy concerns were positively associated with adoption intention (β = 0.230, p < 0.001), suggesting informed and critical engagement rather than resistance. Female students reported higher adoption intention than males (β = 0.137, p = 0.002), while prior publication experience was negatively associated with intention (β = −0.089, p = 0.036). Demographic variables such as age, specialty, and marital status were not significant predictors. The adoption-intention model demonstrated moderate explanatory power (adjusted R^2^ = 0.560).

**Conclusions:**

Among nursing and health profession master’s students at a regional Saudi university, findings indicate pragmatic optimism toward AI integration in academic research, driven primarily by perceived benefits alongside heightened ethical and privacy awareness. Privacy concerns appear to reflect critical literacy rather than barriers to adoption.

## Background

Artificial intelligence (AI) has emerged as a transformative force in higher education and research, fundamentally reshaping how postgraduate students approach scholarly inquiry, data analysis, and knowledge production [1,2]. Generative AI platforms such as ChatGPT have rapidly permeated academic environments, offering capabilities ranging from literature synthesis and data interpretation to code generation and manuscript drafting [1,3]. Concurrently, debates about research credibility in the AI era underscores the need to investigate how students utilize these tools to enhance scholarly outputs while maintaining ethical standards [4]. However, this integration has also sparked urgent debates about academic integrity, research credibility, algorithmic bias, and the potential erosion of critical thinking skills [3]. Understanding how postgraduate students perceive and engage with AI tools is therefore critical for aligning technological adoption with educational objectives, ethical standards, and professional development goals.

The significance of examining AI adoption in postgraduate education is amplified by persistent inequities in access to research resources, particularly in historically disadvantaged universities and developing regions where AI tools may serve as cost-effective alternatives to expensive proprietary databases and extensive library collections [5]. In these contexts, AI integration represents not merely a technological upgrade but a potential equalizer that could bridge resource gaps and enhance research capacity. Yet, without careful consideration of students’ perceptions, including their recognition of benefits, concerns about risks, and awareness of ethical implications, institutional policies risk either uncritical adoption that overlooks legitimate concerns or overly restrictive regulations that stifle innovation [3]. AI’s expanding role in education marks it as a transformative technology, with applications ranging from personalized learning to automated research assistance [6,7]. For instance, in medical education, students’ attitudes toward AI directly influence their readiness to adopt it in clinical practice, necessitating curriculum reforms that address both technical competencies and ethical literacy [3,8]. Similarly, dental students’ perceptions of AI tools reveal gaps in training, highlighting the importance of tailored educational interventions to bridge theoretical knowledge and practical application [8,9].

The shift toward online learning, accelerated by the COVID-19 pandemic, has further intensified reliance on AI-driven platforms [10]. Students now engage in smart classrooms and interactive applications designed to enhance engagement, though disparities in digital access and data privacy concerns persist [11,12]. For example, while AI-powered tools improve feedback efficiency in language education, ethical dilemmas, such as algorithmic bias and data security, demand greater scrutiny to ensure equitable implementation [13,14].

Empirical studies employing cross-sectional surveys highlight nuanced student perspectives. Medical students, for instance, recognize AI’s potential to streamline diagnostics but express apprehension about its ethical implications in patient care [15,16]. Similarly, postgraduate researchers acknowledge AI’s utility in data analysis but stress the need for transparent guidelines to preserve academic integrity [17]. These findings underscore the importance of fostering AI literacy while addressing concerns about over-reliance on automation in scholarly work [18,19]. As AI reshapes professional landscapes, educators face the challenge of preparing students for careers intertwined with intelligent systems. Medical curricula, for example, must evolve to equip future physicians with competencies in AI-driven diagnostics and ethical decision-making [20,21]. Similarly, postgraduate programs must balance technical training with critical discussions about AI’s societal impacts, ensuring graduates contribute meaningfully to research and innovation [22,23].

Postgraduate students’ perceptions of AI tools are shaped primarily by perceived benefits, which consistently emerge as the strongest driver of adoption. Studies show that learners across health, medical, and higher-education disciplines value AI for improving research efficiency, supporting literature synthesis, enhancing writing quality, and simplifying complex analytical tasks [1,3]. These benefits align with the Technology Acceptance Model, where perceived usefulness is the most influential predictor of intention to use digital technologies [24]. At the same time, research indicates that students maintain a pragmatic awareness of AI limitations, acknowledging risks such as inaccurate outputs, hallucinated references, and reduced opportunities to develop independent critical-thinking skills [25,26].

Alongside benefits, students’ express concerns related to privacy, mistrust, ethical risks, performance anxiety, social bias, and lack of regulation, all of which form essential variables in technology-adoption research. Privacy concerns are frequently highlighted, with students fearing data misuse or unclear storage practices, particularly in health-profession programs where confidentiality is critical [15,27]. Mistrust in AI mechanisms, such as opaque algorithms and inconsistent accuracy, also contributes to cautious use, while regulatory and liability concerns reflect uncertainty about ethical boundaries and accountability when AI-generated content is used in academic work [28,29]. Despite these challenges, empirical studies consistently describe a pattern of pragmatic optimism, where students appreciate AI’s value but expect clear guidelines to ensure responsible, ethical, and safe integration into research and learning.

### The Saudi Arabian context and research gap

Saudi Arabia presents a particularly compelling context for investigating AI adoption in postgraduate education due to the nation’s ambitious digital transformation agenda under Vision 2030. This strategic framework prioritizes innovation, knowledge economy development, and technological leadership, driving substantial investments in AI infrastructure, digital literacy initiatives, and smart education platforms [30]. Recent studies document Saudi Arabia’s rapid adoption of generative AI tools, with 78.7% of students reporting frequent use of ChatGPT for tasks such as literature reviews, data analysis, and academic writing. This high adoption rate aligns with national initiatives such as the Saudi Academic Framework for AI Qualifications, which seeks to integrate AI competencies into higher education curricula while establishing ethical guidelines for responsible use. However, challenges persist. Concerns about academic integrity, often termed “AI-giarism”, have intensified as students increasingly rely on AI-generated content for assignments and research outputs [30,31].

Reliability of AI-generated information remains a significant concern, particularly when outputs contain factual errors, outdated references, or biased interpretations. Additionally, disparities in digital access across regions and institutions raise equity concerns, as students in well-resourced urban universities may benefit disproportionately compared to those in regional or under-resourced institutions. Despite these challenges, cross-sectional surveys in Saudi universities reveal that postgraduate researchers recognize AI’s transformative potential while stressing the need for transparent guidelines to preserve scholarly rigor and maintain public trust in research outputs [31]. Cultural and institutional factors uniquely shape Saudi students’ trust in and engagement with AI tools. Unlike Western contexts where individualism and autonomy may drive technology acceptance, collectivist values and institutional authority may play more prominent roles in Saudi educational settings. Furthermore, the rapid pace of AI integration, driven by top-down national policy initiatives, may create tensions between institutional expectations and students’ personal readiness or ethical concerns. These contextual nuances underscore the importance of region-specific research that goes beyond importing technology acceptance models developed in Western contexts.

### Study rationale and research questions

Despite growing global interest in AI adoption in education, significant gaps remain in understanding how postgraduate students in nursing and health professions, disciplines deeply rooted in ethical practice, patient care, and evidence-based decision-making, perceive AI integration in research contexts. Most existing studies focus on undergraduate populations, STEM disciplines, or general learning applications rather than research-specific use cases [1,3]. This study is conceptually grounded in the Technology Acceptance Model (TAM), which posits that technology adoption is primarily driven by perceived usefulness and perceived ease of use. In the context of AI-assisted research, perceived benefits represent perceived usefulness, while concerns such as privacy, mistrust, and performance anxiety reflect perceived risk and uncertainty factors that may shape adoption intention. Rather than testing formal hypotheses, this study adopts an exploratory explanatory approach appropriate for emerging technologies in under-researched contexts.

Furthermore, regional disparities in AI adoption research leave Middle Eastern contexts, including Saudi Arabia, underrepresented in the literature, limiting the generalizability of findings and the relevance of policy recommendations. This study addresses these gaps by investigating postgraduate students’ perceptions of AI integration in research at Northern Border University (NBU), a regional public university in Saudi Arabia. NBU represents a particularly informative case study: as a regional institution operating within the Vision 2030 framework but facing resource constraints typical of non-metropolitan universities, NBU exemplifies the opportunities and challenges of AI adoption in contexts where technological innovation must coexist with limited infrastructure and support systems. By focusing on nursing and health profession master’s students, populations whose professional identities are deeply connected to ethical practice and patient welfare, this study illuminates how disciplinary values intersect with technological adoption.

The study was guided by three research questions:

1. **RQ1:** How fa`miliar are postgraduate students with AI tools like ChatGPT, and how frequently do they incorporate them into their research practices?
2. **RQ2:** What are postgraduate students’ perceptions of the benefits and potential drawbacks of using AI to enhance research efficiency and quality?
3. **RQ3:** What concerns do postgraduate students have regarding performance anxiety, social bias, privacy, trust, & moral challenges that come with using AI tools in their research?

### Conceptual definitions of study variables

The study examined ten AI perception dimensions that have been widely discussed in the literature on technology acceptance, AI ethics, and digital trust. Perceived performance anxiety reflects concerns about AI reliability and fear of over-dependence on automated systems. Perceived social biases relate to awareness of algorithmic bias and fairness issues. Privacy concerns capturing apprehension about data security, confidentiality, and unauthorized access. Mistrust in AI mechanisms reflects skepticism toward algorithmic transparency and explainability. Communication barriers refer to difficulties interpreting AI outputs or integrating them into academic communication [15,27,29,30]. Lack of standardized regulations reflects concerns about insufficient institutional or ethical guidelines governing AI use. Liability issues relate to uncertainty about responsibility for AI-generated errors. Perceived risks represent overall concerns about negative consequences of AI integration, while perceived benefits reflect beliefs about AI’s usefulness in enhancing research efficiency and quality [24,27]. Intention to adopt AI tools represents willingness to integrate AI into current and future research practices. These constructs informed the regression model presented in Table 4.

**Table 4:** Multiple linear regression predicting intention to adopt AI-based tools.

Predictor	B	SE	95% CI	β (std)	p
**Intercept**	−0.038	0.415	[-0.851, 0.774]		0.926
**Demographics**					
**Age (per year)**	0.002	0.007	[-0.011, 0.015]	0.013	0.78
**Sex (Female vs. Male)**	0.216	0.071	[0.077, 0.354]	.137**	0.002
**Specialty (Nursing vs. Other Health)**	0.079	0.081	[-0.079, 0.237]	0.049	0.329
**Marital Status (Married vs. Single)**	0.118	0.071	[-0.021, 0.257]	0.074	0.095
**Experiential Factors**					
**Currently Involved in Research**	0.265	0.092	[0.084, 0.446]	.145**	0.004
**Completed Prior Research Projects**	0.129	0.089	[-0.045, 0.302]	0.077	0.147
**Prior Publication(s)**	−0.151	0.072	[-0.292, -0.010]	−.089*	0.036
**Prior AI Tool Use (Yes vs. No)**	0.39	0.086	[0.222, 0.559]	.178***	<.001
**Perception Dimensions**					
**Performance Anxiety**	−0.054	0.072	[-0.195, 0.087]	−0.058	0.457
**Social Biases**	−0.082	0.101	[-0.280, 0.116]	−0.085	0.415
**Privacy Concerns**	0.207	0.053	[0.104, 0.311]	.230***	<.001
**AI skepticism**	0.118	0.065	[-0.009, 0.245]	0.121	0.069
**Communication Barriers**	−0.035	0.076	[-0.183, 0.113]	−0.041	0.644
**Undefined guidelines**	−0.011	0.089	[-0.186, 0.163]	−0.012	0.901
**Accountability concerns**	0.024	0.093	[-0.158, 0.205]	0.026	0.798
**Perceived Risks**	−0.1	0.058	[-0.213, 0.014]	−0.105	0.085
**Perceived Benefits**	0.584	0.062	[0.463, 0.704]	.588*	<.001

Model Fit: n = 265 (2 cases excluded due to missing data), Adjusted R² = .560, AIC = 425.3, F (18, 246) = 19.84, p < .001. Diagnostics: VIF range 1.2–4.8 (acceptable multicollinearity), no influential outliers (Cook’s D < 0.15), heteroscedasticity-consistent (HC3) robust standard errors applied. ***p < .001; **p < .01; *p < .05

## Methodology

### Study design

This study employed a cross-sectional survey design to examine postgraduate students’ perceptions of artificial intelligence (AI) integration in academic research. The cross-sectional approach was selected for its efficiency in capturing perceptions, attitudes, and behavioral intentions at a single time point, providing a foundational understanding of AI adoption patterns within the target population [24]. However, we acknowledge that this design precludes causal inference and cannot establish temporal precedence between predictors and outcomes. Additionally, cross-sectional data captures behavioral intentions rather than actual behavior, necessitating caution when generalizing findings to sustain AI use patterns. Future longitudinal research tracking the same cohort over time would be valuable for understanding how perceptions and usage evolve as AI technologies mature and improve students’ progress through their research training.

### Study setting and context

This study was conducted at Northern Border University (NBU), a regional public university located in Arar, Saudi Arabia. Established in 2007 as part of Saudi Arabia’s higher education expansion initiative, NBU serves the Northern Border Region and operates within the framework of Vision 2030, the nation’s strategic plan emphasizing digital transformation, innovation, and knowledge economy development [30]. As a regional institution, NBU faces resource constraints typical of non-metropolitan universities, including limited access to proprietary research databases, restricted physical library collections, and fewer opportunities for face-to-face research mentorship compared to larger urban institutions. In this context, AI tools may represent particularly attractive alternatives as cost-effective research support mechanisms. The institutional environment at NBU actively encourages digital literacy and technological integration, aligning with national policy priorities that normalize AI adoption in academic settings.

### Definite population definition

The target population for this study comprised comprises all master’s-level students enrolled in nursing and health profession graduate programs at Northern Border University (NBU) during the 2025 academic year. While the exact total number of eligible students is not publicly reported by the institution, the study recruited 267 participants, which represents the full accessible population reached through university-wide invitations. Accordingly, this study constitutes a single-institution case study situated within a regional Saudi university operating under the national Vision 2030 digital transformation framework. Findings are therefore not intended to represent postgraduate students nationally or across disciplines, but rather to provide context-specific evidence from a health-profession cohort within one institution.

### Sample size determination and recruitment

Sample size was calculated a priori using G*Power 3.1 software for multiple linear regression analysis. The calculation assumed 18 predictors (demographic variables, experiential factors, and perception dimensions), a medium effect size (f^2^ = 0.15) based on prior technology acceptance literature [24], alpha level α = 0.05, and desired statistical power of 0.80. These parameters yielded a minimum required sample size of 179 participants. To account for potential incomplete responses, non-engagement, and attrition, we aimed to recruit 267 participants, providing a buffer of approximately 49% above the minimum requirement and ensuring adequate statistical power for detecting meaningful associations.

Participants were recruited through convenience sampling from all master’s-level programs in nursing and health professions across colleges affiliated with Northern Border University. Convenience sampling was selected for its feasibility given the target population’s dense academic schedules, geographic dispersion across multiple campuses, and time constraints associated with data collection during the academic semester. While this approach ensured accessibility and practical recruitment efficiency, it introduces sampling bias favoring more accessible, motivated, and technologically engaged students. Consequently, findings may not represent AI-novice populations or students with limited digital access or negative attitudes toward technology.

Recruitment strategies included: (a) direct invitation by the research team during scheduled postgraduate lectures, (b) distribution of the survey link via university email to all registered master’s students in eligible programs, (c) dissemination through social media platforms commonly used by students (WhatsApp, Twitter/X), and (d) sharing the survey link with faculty teaching in postgraduate programs, who forwarded invitations to their students. Weekly reminders were issued over the six-week data collection period (October 1 to November 15, 2025) to maximize response rates. A final sample of 267 nursing and health profession postgraduate students was successfully recruited.

The study included participants who are currently enrolled as master’s students in nursing or other health-related programs at Northern Border University, who are willing to provide informed consent, and who have prior experience using or evaluating AI tools, such as ChatGPT, DeepSeek, Zotero, QuillBot, Grammarly, or Mendeley, for research purposes including literature searches, data analysis, citation management, writing support, or conceptual exploration. Individuals will be excluded if they are undergraduate or doctoral (PhD) students, faculty members, non-degree-seeking researchers, or students enrolled in non-health disciplines such as business, education, or engineering. Students who decline to provide informed consent will also be excluded.

### Data collection tools

Data was collected using a validated self-administered questionnaire adapted from Esmaeilzadeh (2020) [32], who originally developed the instrument to assess perceptions of AI risks and benefits in nursing research contexts. The instrument was administered electronically via Google Forms to ensure accessibility, anonymity, and efficient data management. The questionnaire comprised three main sections:

### Section 1: Demographic and Background Characteristics

This section collected information on age, sex, marital status, master’s program specialty (nursing vs. other health professions), research experience (currently involved in research, completed prior research projects, prior publications), and prior AI tool use (yes/no, with specification of tools used).

### Section 2: AI Perception Dimensions

This section consisted of 54 items distributed across ten subscales, each employing a five-point Likert scale ranging from 1 (*strongly disagree*) to 5 (*strongly agree*). The ten subscales assessed:

1. **Perceived Performance Anxiety** (5 items): Concerns about AI reliability, fear of technological dependence, and anxiety about AI replacing human judgment in research tasks
2. **Perceived Social Biases** (5 items): Awareness of algorithmic bias, fairness concerns, and potential for AI to perpetuate or amplify existing social inequities in research contexts
3. **Perceived Privacy Concerns** (6 items): Apprehensions about data security, confidentiality breaches, unauthorized data access, and institutional misuse of personal or research data
4. **Perceived Mistrust in AI Mechanisms** (5 items): Skepticism about AI transparency, algorithmic explainability, and confidence in AI-generated outputs
5. **Perceived Communication Barriers** (5 items): Difficulties in interpreting AI outputs, challenges in communicating AI-assisted findings, and concerns about AI’s ability to understand context or nuance
6. **Perceived Lack of Standardized Regulations** (5 items): Concerns about insufficient institutional policies, absence of ethical guidelines, and regulatory gaps governing AI use in academic research
7. **Perceived Liability Issues** (6 items): Uncertainty about responsibility for AI errors, legal accountability for AI-generated content, and intellectual property concerns
8. **Perceived Risks** (5 items): Overall assessment of risks associated with AI integration, rated from *very low* to *very high*
9. **Perceived Benefits** (7 items): Recognition of AI’s potential to enhance research efficiency, improve data analysis quality, facilitate literature synthesis, accelerate discovery, and democratize access to research tools
10. **Intention to Adopt AI-Based Tools** (5 items): Willingness to integrate AI tools into current and future research practices, openness to learning new AI applications, and likelihood of recommending AI tools to peers

**Section 3: Overall AI Perception:** An overarching summary question asked: “Considering all factors, how do you perceive the use of AI in nursing and health profession research?” Response options ranged from *very negative* to *very positive* on a five-point scale. An overall AI perception score was computed by aggregating responses across all 54 items and the summary question.

### Instrument validation and reliability

The original instrument developed by Esmaeilzadeh (2020) demonstrated strong psychometric properties. Convergent validity was established through standardized factor loadings, composite reliability coefficients, and average variance extracted (AVE) values exceeding recommended thresholds. Discriminant validity was confirmed, as the square root of each construct’s AVE exceeded 0.70 and surpassed intercorrelations with other constructs, indicating that each subscale measured a distinct dimension. Internal consistency reliability was assessed using Cronbach’s alpha coefficients. The overall instrument demonstrated excellent reliability (α = 0.964). Subscale-specific reliability coefficients were as follows: perceived benefits (α = 0.940), perceived risks (α = 0.900), performance anxiety (α = 0.910), social biases (α = 0.880), privacy concerns (α = 0.859), mistrust in AI mechanisms (α = 0.826), communication barriers (α = 0.869), lack of standardized regulations (α = 0.846), liability issues (α = 0.862), and intention to adopt AI-based tools (α = 0.897). All subscales exceeded the conventional threshold of α = 0.70, indicating acceptable to excellent internal consistency.

### Instrument adaptation and translation

The study utilized the original English version of the instrument developed by Esmaeilzadeh (2020) without cultural or linguistic adaptation. This decision was appropriate because all participants were postgraduate students in health sciences, a group for whom English is the primary language of academic instruction and research engagement. Therefore, no translation or back‑translation procedures were required. The instrument’s established psychometric properties, including its construct validity and reliability, were drawn from the original development study. In the present study, internal consistency reliability (Cronbach’s alpha) was recalculated for each subscale to confirm acceptable reliability within the current sample; however, additional validity testing was not conducted, as the aim was to employ the tool as originally designed rather than redevelop or revalidate

### Ethical considerations

The study adhered to the principles outlined in the “Declaration of Helsinki.” Ethical agreement was granted from Northern Border University with the following number (HAP-09-A-043) with decision number (74/25/H) 30-9-2025. Electronically informed consent was obtained from all participants before accessing the questionnaire, with the consent form outlining the study’s purpose, procedures, risks, benefits, confidentiality measures, and the voluntary nature of participation. Participants who selected “I agree to participate” proceeded to the survey, while those who declined were redirected to a thank-you page. Confidentiality was maintained by collecting data anonymously and storing responses on password-protected Google servers accessible only to the research team. Data was later transferred to encrypted, password-protected devices for analysis and will be securely archived for at least five years in accordance with institutional policy. The study involved minimal risk, limited to potential mild discomfort when reflecting on personal views about AI, and no deception, interventions, or invasive procedures were used. Participation was completely voluntary, and students were assured that choosing not to participate, or withdrawing at any point by closing the survey, would not affect their academic standing or access to university services; however, submitted responses could not be withdrawn due to the anonymity of the data.

### Statistical analysis

All analyses were performed using IBM SPSS Statistics version 28.0, with statistical significance set at α = 0.05. Descriptive statistics summarized demographic characteristics, AI usage patterns, and perception scores, with categorical variables reported as frequencies and percentages, and continuous variables assessed for normality using histograms, Q–Q plots, and the Shapiro–Wilk test. Age demonstrated a positively skewed distribution (Mean = 28.9, SD = 5.2; Median = 29, IQR = 26–33; skewness = 1.84; range = 20–52). The discrepancy between the mean and median reflects the influence of older non‑traditional students, and data verification procedures were implemented to correct inconsistencies identified during quality checks. Two multiple linear regression models were constructed: Model 1 examined predictors of intention to adopt AI‑based tools using demographic variables, research experience, prior AI use, and nine AI perception dimensions; Model 2 assessed predictors of overall AI perception using the same variables. Diagnostic checks showed acceptable multicollinearity (VIF 1.2–4.8), approximately normal residuals, and generally homoscedastic variance; robust HC3 standard errors were applied to address minor heteroscedasticity. No influential outliers were detected using Cook’s distance or leverage values. Missing data was minimal (<1%) and handled using listwise deletion. Model fit was evaluated using adjusted R^2^ and AIC, with Model 1 showing moderate fit (adjusted R^2^ = 0.560; AIC = 425.3) and Model 2 showing an extremely high adjusted R^2^ (0.993) due to methodological circularity, as the dependent variable was derived from component predictors. Thus, Model 2 serves primarily as a structural validity check for the perception instrument, while the substantive findings of the study are derived from Model 1, which assesses adoption intention based on distinct theoretical predictors.

## Results

### RQ1: AI tool familiarity and incorporation into research practices

Table 1 Participant Characteristics and Prior AI Exposure. This table presents demographic and experiential characteristics of the 267 nursing and health profession master’s students who participated in this single-institution study at Northern Border University between October 1 and November 15, 2025. This sample represents a specific subpopulation: postgraduate students at a regional Saudi university with high prior AI exposure, rather than the broader postgraduate education landscape. Among the participants who reported prior AI use (85.0%), the most used tool was ChatGPT, used by 221 participants (97.4%). Writing-support tools were also widely adopted, including Grammarly (68.7%) and Quill Bot (59.0%). Reference management tools with AI capabilities were used less frequently, with 39.2% reporting Zotero with AI plugins and 33.5% using Mendeley. DeepSeek, used as a research assistant, was reported by 15.0% of participants, while 12.3% used other AI tools such as Perplexity, Elicit, and Consensus. Most participants (85.0%) had prior experience with AI tools, predominantly ChatGPT, indicating that this sample represents technologically engaged, AI-familiar students rather than novice users.

**Table 1:** Personal characteristics of study participants (N = 267).

Characteristic	n (%) or Statistic
**Age (years)**	
Mean (SD)	28.9 (5.2)
Median (IQR)	29 (26–33)
Range	20–52
Skewness	1.84
**Gender**	
Female	142 (53.2)
Male	125 (46.8)
**Master’s Specialty**	
Nursing	130 (48.7)
Other Health Professions ‡	137 (51.3)
**Marital Status**	
Married	155 (58.1)
Single	112 (41.9)
**Prior AI Tool Use**	
Yes	227 (85.0)
No	40 (15.0)
**Research Experience**	
Currently involved in research	148 (55.4)
Completed prior research project	177 (66.3)
Prior publication(s)	82 (30.7)

‡ Other health professions include public health, health administration, clinical nutrition, and rehabilitation sciences.

### RQ2: Perceptions of AI benefits and drawbacks

Table 2 presents mean scores and internal consistency reliability (Cronbach’s α) for each of the ten perception dimensions assessed by the 54-item instrument. All dimensions demonstrated acceptable to excellent reliability (α = 0.826–0.964). Participants rated perceived benefits highest (M = 4.12, SD = 0.68), indicating strong recognition of AI’s potential to enhance research efficiency, improve literature synthesis, accelerate data analysis, and democratize access to sophisticated research tools. Adoption intention was moderately high (M = 3.89, SD = 0.82), suggesting pragmatic openness to integrating AI into research practices.

**Table 2:** Descriptive statistics and reliability of AI perception dimensions.

Dimension	No. Items	Mean (SD)	Range	Cronbach’s α
**Perceived Benefits**	7	4.12 (0.68)	1.71–5.00	0.94
**Adoption Intention**	5	3.89 (0.82)	1.20–5.00	0.897
**Privacy Concerns**	6	3.76 (0.74)	1.50–5.00	0.859
**Mistrust in AI Mechanisms**	5	3.42 (0.81)	1.20–5.00	0.826
**Performance Anxiety**	5	3.38 (0.88)	1.00–5.00	0.91
**Liability Issues**	6	3.35 (0.79)	1.33–5.00	0.862
**Lack of Standardized Regulations**	5	3.31 (0.84)	1.00–5.00	0.846
**Communication Barriers**	5	3.18 (0.91)	1.00–5.00	0.869
**Social Biases**	5	3.12 (0.87)	1.00–5.00	0.88
**Perceived Risks (overall)**	5	3.08 (0.79)	1.00–4.80	0.9
**Overall, AI Perception Score**	54	3.56 (0.58)	2.15–4.87	0.964

Notes: All items rated on 5-point Likert scales (1 = strongly disagree, 5 = strongly agree). Higher scores indicate greater endorsement of the dimension. Overall, AI Perception Score computed as the mean across all 54 items.

Participants simultaneously expressed substantial concerns across several dimensions. Privacy concerns were the most prominent (M = 3.76), reflecting apprehensions about data security, confidentiality breaches, and unauthorized access to research data. Mistrust in AI mechanisms (M = 3.42) was also evident, with participants questioning the transparency, explainability, and reliability of AI-generated outputs. Performance anxiety (M = 3.38) emerged as another key issue, including worries about over-reliance on AI, dependence on technology, and the potential replacement of human judgment. Concerns related to liability (M = 3.35) highlighted uncertainty about responsibility for AI errors, intellectual property implications, and legal accountability. Additionally, participants noted the lack of standardized regulations (M = 3.31), citing insufficient institutional policies and ethical guidelines governing AI use.

### RQ3: Concerns regarding ethical implications, privacy, trust, and social biases

To assess whether ethical concerns (performance anxiety, social biases, privacy, mistrust) represent distinct constructions or a unified “skepticism” factor, we examined Pearson correlations among challenge dimensions (Table 3). Moderate-to-strong positive correlations (r = .49–.71) among challenge dimensions indicate that students who express concerns in one area (e.g., privacy) tend to express concerns in others (e.g., mistrust, liability). This suggests critical awareness: students with a more sophisticated understanding of AI’s complexities recognize interconnected ethical challenges rather than focusing narrowly on single issues. Importantly, these correlations are not near perfect (all r < .75), confirming that each dimension captures distinct aspects of AI concern rather than redundant measurements of a single “anti-AI sentiment.”

**Table 3:** Intercorrelations among AI challenge dimensions.

Dimension	1	2	3	4	5	6	7	8
**1. Performance Anxiety**								
**2. Social Biases**	.62**							
**3. Privacy Concerns**	.54**	.58**						
**4. Mistrust in AI**	.59**	.61**	.68**					
**5.Communication Barriers**	.57**	.64**	.52**	.63**				
**6. Unregulated Standards**	.51**	.59**	.61**	.64**	.60**			
**7. Liability Issues**	.49**	.55**	.67**	.62**	.56**	.71**		
**8. Perceived Risks**	.63**	.66**	.61**	.68**	.61**	.65**	.64**	

Note: **p < .01. All correlations significant at p < .001.

**Fig 1:**
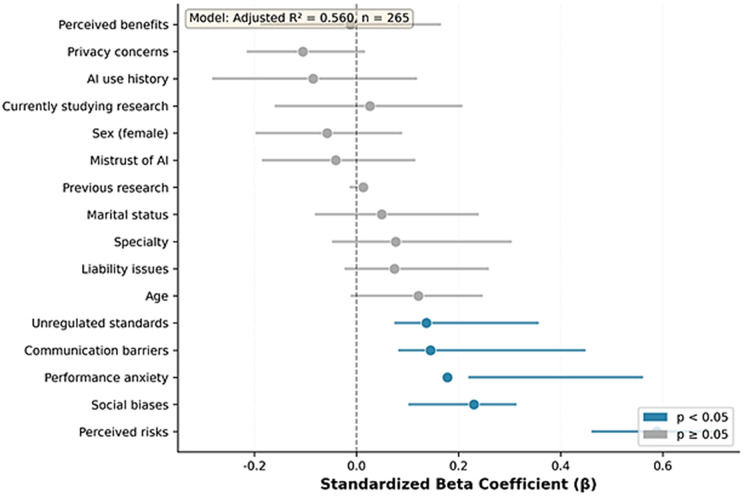
Forest plot of standardized predictors.

**Fig 2:**
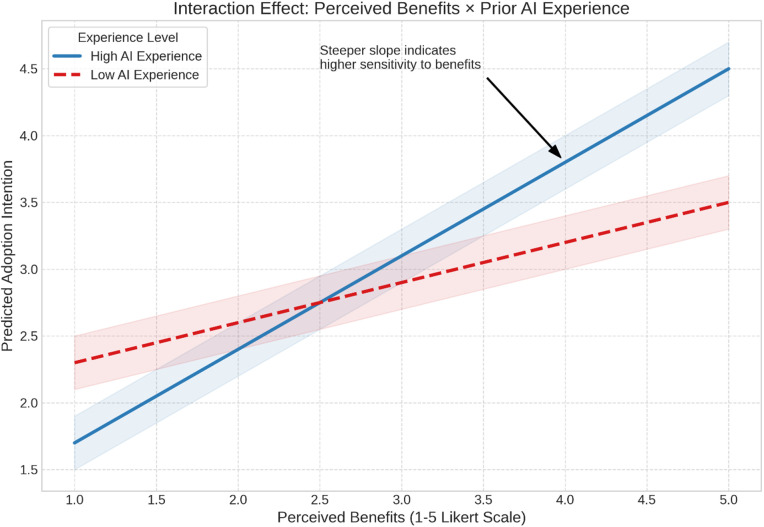
Predictive Margins - Benefits × AI Experience.

**Fig 3:**
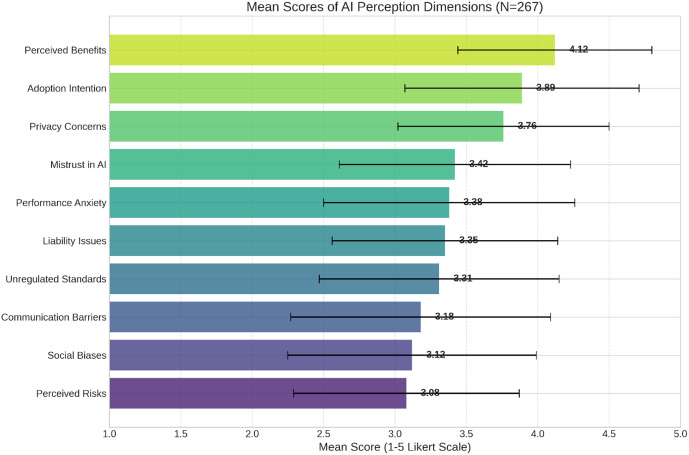
Cross-model comparison.

Table 4 illustrates Multiple Linear Regression Predicting Intention to Adopt AI-Based Tools. The regression findings indicate that perceived benefits were by far the strongest predictor of AI adoption intention, with higher perceived usefulness, such as increased efficiency and improved research performance, driving substantially greater willingness to adopt AI tools. In contrast, privacy concerns showed a surprising positive association with adoption, suggesting that students who are more aware of data-security issues may also be more digitally literate and confident in using AI responsibly. Female students demonstrated higher adoption intention than males, likely reflecting the disciplinary context of nursing and health sciences, where AI may be viewed as a supportive tool that enhances research confidence. Conversely, students with prior publication experience were less inclined to adopt AI, a pattern linked to higher mistrust in AI mechanisms and greater familiarity with research standards that make experienced researchers more cautious about AI reliability. Finally, experiential familiarity, both prior AI use and active research engagement, significantly increased adoption intention, underscoring that direct experience reduces uncertainty and fosters comfort with AI tools, while demographic variables and most challenge dimensions showed no meaningful explanatory power Figs 1–3.

## Discussion

The integration of artificial intelligence (AI) into academic processes is rapidly transforming research and education, making the study of its adoption among postgraduate students a critical area for educational institutions and policymakers. Research attention has thus turned into popular tools like ChatGPT and their role in academic environments [33,34]. ChatGPT is recognized for its transformational potential. It functions as a dynamic aid for researchers, supporting activities ranging from brainstorming and drafting research paper components to generating code [35]. This study investigated the perceptions of postgraduate students in nursing, medical and health sciences at Northern Border University (NBU) in Saudi Arabia regarding the integration of AI tools, focusing on perceived benefits, practical challenges, and ethical implications. Our findings offer empirical evidence that aligns with and extends established technology acceptance theories while providing context-specific insights relevant to the rapidly evolving Saudi higher education landscape.

The context of NBU, a regional public university established under the umbrella of Saudi Vision 2030, is crucial for interpreting our results. Vision 2030 has driven significant national investment in digital transformation and innovation, creating an institutional environment that normalizes and encourages AI integration [36]. For a regional university like NBU, which may face resource constraints compared to larger metropolitan institutions, AI tools can be particularly attractive as cost-effective alternatives to proprietary databases or extensive physical library collections. This national and institutional support likely amplifies students’ willingness to engage with AI, a factor that must be considered when assessing the generalizability of our findings. Consequently, findings should be interpreted as reflecting perceptions among nursing and health profession master’s students at Northern Border University with prior AI exposure, rather than postgraduate students broadly across all disciplines, institutions, or geographic regions in Saudi Arabia or internationally.

### RQ1: Familiarity with AI tools and integration into research practices

Addressing the first research question regarding familiarity and frequency of AI tool incorporation, current study results demonstrated widespread prior exposure to artificial intelligence tools among participants, with ChatGPT emerging as the predominant platform. This commonality points to a substantial level of AI integration in postgraduate medical training, implying that existing familiarity is likely to influence subsequent patterns of adoption and application. These findings align with international contexts; for instance, Liu et al., (2024) [37] observed that Chinese nursing students employed ChatGPT to complete homework and essays, thereby enriching their educational experience. Likewise, Pallivathukal et al., (2024) [38] documented its use among healthcare students in Malaysia for conceptual understanding and question generation. Similarly, Owan, et al., (2025) [39] reported in their study that postgraduate students demonstrated a strong willingness to use AI for their independent research learning, scoring well above the benchmark.

This acceptance is consistent with Technology Acceptance Model (TAM) core idea that the adoption of a new technology is driven by how useful and easy to use it is perceived to be. The students’ high level of acceptance was specifically shown by their eagerness to learn advanced research methods with AI and their view of it as a trustworthy supplement to conventional guidance, both of which indicate a strong perception of its usefulness as a key factor in accepting educational technology. However, familiarity and behavioral intention do not necessarily translate to actual sustained usage behavior. Future longitudinal studies are needed to determine whether the adoption intentions documented here materialize into consistent, long-term AI integration in research workflows, or whether initial enthusiasm diminishes as students encounter practical challenges or complete their programs.

### Gender Differences in AI Adoption Intention: A Counterintuitive Finding Requiring Deeper Exploration

A key finding in the current study from the multiple regression was that gender significantly influenced the intention to adopt AI, with female students demonstrating a stronger inclination to use these tools than males. The positive beta value (β = 0.137) indicates a measurable, positive relationship, and the high level of statistical significance (p = 0.002) gives high confidence in this result. This gender effect diverges from some prior literature and warrants deeper mechanistic exploration. On contrary, the recent studies conducted by (Akabar, 2024; Iddrisu et al., 2025) [40,41], reported that no significant gender gap in general AI attitudes. The discrepancy highlights a critical distinction in technology acceptance: while men and women might hold similar broad views about AI, their willingness to integrate it into their work or studies can differ.

Several interpretations may explain why female students in this sample reported stronger intentions to adopt AI tools. First, because the sample is predominantly drawn from nursing and other health‑related disciplines fields with a high proportion of women gender may be acting as a proxy for disciplinary culture rather than reflecting a true gender-based difference. Female students in nursing programs may view AI as a valuable supplement to research‑methods training, which traditionally receives less emphasis in clinically oriented curricula compared with STEM fields. Second, the institutional and sociocultural environment is relevant. Within the Saudi higher education context at NBU, where Vision 2030 initiatives actively promote women’s academic advancement and technological empowerment, female students may perceive AI adoption as aligned with national expectations of professional modernization. Third, confidence-related mechanisms may also contribute. If female students feel less assured in conventional quantitative research methods due to earlier educational experiences, AI tools that automate statistical procedures or literature synthesis may help reduce anxiety and lower perceived barriers to engaging in research tasks.

### RQ2: Perceived benefits as the dominant driver of adoption intention

In direct response to the second research question concerning perceptions of benefits and drawbacks, the current study revealed that perceived benefits were the most significant predictors (β = 0.588, p < 0.001), highlighting the pivotal influence of perceived usefulness on individuals’ intentions to adopt new technologies. In alignment with this, Algunmeeyn and Mrayyan (2025) [24] found that the average perception of AI’s benefits in scientific research was notably high. This indicates that nursing students recognize AI’s potential to substantially improve the research process. According to Mehdipour (2019) [42], artificial intelligence has the potential to simplify complex tasks, analyze large volumes of data, and detect patterns that might be missed by human observers. These strengths can significantly improve research precision and productivity, leading to enhanced patient care and clinical outcomes, as highlighted by Han. (2025) [29].

As hypothesized, Perceived Benefits emerged as the most influential predictor, reflected in a markedly high standardized coefficient that stood out prominently against other factors in the analysis. This finding is consistent with Zhang et al. (2025) [43], who reported that postgraduate nursing students generally held favorable attitudes toward AI. They valued its capacity to manage data and process language and images effectively, which in turn enhanced their efficiency in academic work. Furthermore, the prominent influence of perceived benefits is further supported by Algunmeeyn and Mrayyan (2025) [24], who reported a high mean score for this construct among nursing students engaged in scientific research. This result shows that students truly feel AI can play a major role in improving the research process. They specifically acknowledge AI’s ability to handle repetitive tasks, process extensive datasets, and uncover intricate patterns that humans might overlook. (Mehdipour, 2019) [42].

### RQ3: Privacy concerns as markers of informed awareness, not barriers to adoption

Addressing the third research question regarding concerns about performance anxiety, social bias, privacy, trust, and ethical implications. In this study, both privacy concerns (β = 0.192, p < 0.001) and liability considerations (β = 0.162, p < 0.001) significantly influenced students’ perceptions, suggesting that a deeper understanding of ethical and legal implications enhances overall evaluation of AI tools. Notably, the positive effect of privacy concerns is both valuable and counterintuitive: instead of deterring adoption, heightened awareness of data‑protection issues predicted stronger intentions to use AI. This pattern implies that students who are more conscious of privacy risks engage with AI more thoughtfully, approaching it as informed and critical users rather than naïve adopters or outright rejectors. In this context, privacy concerns reflect critical engagement rather than technological resistance. Similarly, Algunmeeyn and Mrayyan (2025) [24] observed that privacy emerged as the most prominent concern, primarily due to AI technologies enabling research institutions to collect vast amounts of personal data from individuals. These issues highlight important ethical and legal concerns about safeguarding data and the risk of mishandling sensitive information. Additional scholars have raised issues surrounding privacy, data security, and ethical considerations, stressing the need for robust data protection measures, transparency, and awareness of GenAI’s broader societal implications (Eacersall, 2024) [44]. Additionally, several studies reported ethical concern associated with AI use (Owan, et al., (2025); Chan, & Hu, (2023) and Farrokhnia, et al., (2024) [25,39,45] showing that postgraduate learners are often skeptical of AI’s reliability and worry about ethical dilemmas like data privacy and academic integrity. This apprehension appears to be a key reason they limit AI to an auxiliary function, avoiding its use for independently learning complex research methods. Developing students’ capacity for critical evaluation becomes increasingly essential as AI becomes more integrated into educational settings. Institutions should avoid viewing privacy‑aware students as impediments to AI adoption; instead, their heightened sensitivity to ethical and data‑protection issues positions them as ideal contributors to shaping best‑practice guidelines and responsible AI governance. Their awareness equips them to lead efforts toward ethical and informed AI integration

The findings of the current study revealed that all perception-related dimensions such as mistrust in AI systems, communication challenges, lack of regulatory frameworks, performance-related stress, perceived risks, and social biases were significantly and positively correlated. This suggests that higher scores in these areas reflect nuanced and well-developed perspectives rather than simplistic judgments. This pattern indicates that higher scores across concern dimensions reflect nuanced, well-developed perspectives rather than simplistic judgments. Students who score high on privacy, mistrust, and bias concerns are not technophobes; they are critical thinkers who recognize AI’s complexity. This reframes ethical concerns as markers of analytical maturity rather than adoption barriers. These results are consistent with Alkhayat et al. (2025), [30] who emphasized the importance of addressing key concerns to optimize AI utilization. Specifically, they noted that AI carries a 23.6% risk of errors and bias, may oversimplify complex topics resulting in shallow comprehension, and could foster dependency that diminishes critical thinking and problem-solving skills. Additionally, AI systems may disseminate inaccurate or biased information and potentially impair interpersonal and communication abilities. Likewise, Pallivathukal et al. (2024) [38] found that undergraduate healthcare students voiced apprehensions regarding the accuracy of data, risks of plagiarism, and growing reliance on ChatGPT for academic tasks. Similarly, Topaz et al. (2025) [26] highlighted key challenges associated with GenAI use among students, particularly the potential for plagiarism and the dissemination of misleading information. Echoing these concerns, Han et al. (2025) [29] reported that participants were wary of plagiarism and noted that GenAI occasionally delivers outdated or inaccurate content, which may hinder its overall utility.

A noteworthy and counterintuitive finding in this study is that prior publication experience negatively predicted AI adoption intention. Several mechanisms may explain this pattern. First, students with publication experience often possess higher standards for methodological rigor and may therefore be more critical of AI‑generated content, particularly given concerns about transparency, accuracy, and traceability of sources. This aligns with recent research showing that experienced researchers tend to exhibit greater skepticism toward generative AI due to perceived risks of factual errors and unreliable reasoning [46,47]. Second, publication experience may correlate with age or career stage, suggesting a possible mediating effect; older or more established researchers may have well‑developed research workflows and feel less need for AI augmentation, or may perceive AI as potentially undermining their hard‑earned expertise. Third, cognitive investment or “sunk‑cost” bias may play a role, as individuals who have invested substantial effort in mastering traditional research skills (manual literature synthesis, statistical software, academic writing conventions) may be less inclined to adopt tools that appear to shortcut these competencies. Future research should examine whether age mediates this relationship, whether published researchers systematically rate AI reliability or trustworthiness lower than their non‑published peers, and whether qualitative data reveal concerns about professional identity or perceived threats to expertise.

### Limitations and recommendations for future research

This study has several limitations that should be considered when interpreting the findings. First, as this study represents a single-institution case study at Northern Border University in Saudi Arabia, limiting generalizability to other universities, regions, disciplines, or national contexts. The findings should not be extrapolated to represent postgraduate students broadly across Saudi Arabia or internationally but rather understood as reflecting perceptions among nursing and health profession master’s students at a regional Saudi university operating under Vision 2030 policy frameworks. Future research should include multi‑institutional samples and diverse academic fields to improve external validity. Second, the use of convenience sampling resulted in a sample largely composed of students already familiar with AI tools, particularly ChatGPT. This may overestimate positive perceptions and adoption intentions. Future studies should adopt more representative sampling strategies that include students with varying levels of technological experience and access.

Third, the cross-sectional design limits causal interpretation and cannot determine whether intentions translate into actual AI use over time. Longitudinal studies are needed to examine how perceptions and behaviors evolve as students’ progress academically and as AI technologies mature. Fourth, reliance on self‑reported data introduces social desirability bias and may not accurately reflect real usage behaviors. Future research should incorporate objective measures such as system usage logs or performance analytics to strengthen the validity of findings. Fifth, the study focused on students in nursing and health-related fields, whose ethical and practice-oriented considerations may differ from other disciplines. Cross‑disciplinary comparative studies are recommended to identify shared versus discipline-specific determinants of AI acceptance. Finally, the study did not explore additional social and organizational factors such as peer influence, supervisor attitudes, or institutional support. Incorporating these variables, along with qualitative methods like interviews or focus groups, would provide deeper insights into the contextual factors shaping AI adoption in postgraduate research settings.

## Conclusion& recommendation

In conclusion, this study aimed to describe and explain perceptions of AI integration among a specific group of NBU postgraduate nursing and health professions students, rather than to optimize outcomes or generalizing broadly. The findings reveal strong familiarity with AI and generally positive adoption intentions, driven primarily by perceived usefulness in enhancing research efficiency. Students also demonstrated informed awareness of challenges such as data privacy, ethical liability, algorithmic bias, and risks of academic misconduct; notably, privacy concerns reflect ethical engagement rather than resistance. The unexpected negative association with prior publication experience suggests that experienced researchers may require tailored support addressing rigor and quality standards. Prior AI exposure and active research involvement positively influenced adoption intention, while demographic factors played a minimal role. The higher adoption intention among female students warrants further exploration.

Overall, the cohort appears both optimistic and critically reflective, recognizing AI’s value while emphasizing the need for clear guidelines and ethical safeguards. Because intention does not ensure sustained behavior, longitudinal follow‑up is needed to determine whether these intentions translate into long‑term research practices. The study recommends that higher education institutions develop transparent policies for responsible AI use, focusing on ethical guidelines, review protocols, and discipline‑specific models rather than universal literacy modules. Training should be differentiated: foundational support for novices and advanced workshops on validation, quality assurance, and publication standards for experienced researchers. Curricula should cultivate critical evaluation skills and ethical reasoning, positioning AI literacy as ongoing professional development. Privacy‑aware students should be engaged as partners in shaping institutional policies. Future longitudinal and multi‑institutional research is needed to track evolving perceptions and to distinguish universal acceptance patterns from context‑specific influences related to institutional resources, national policies, and disciplinary cultures.
